# Supplementary material for: External trigeminal nerve stimulation in youth with ADHD: a randomized, sham-controlled, phase 2b trial
Source: Nat Med. 2026 Jan 16;32(2):582–90. doi: 10.1038/s41591-025-04075-x (PMC12920111; doi:10.1038/s41591-025-04075-x)
Supplement: Supplementary file 1 — Supplementary Tables 1−10. [file 41591_2025_4075_MOESM1_ESM.pdf]

nature medicine

Supplementary information

https://doi.org/10.1038/s41591-025-04075-x

# External trigeminal nerve stimulation in youth with ADHD: a randomized, sham-controlled, phase 2b trial

In the format provided by the authors and unedited

## Table of Contents

Statistical analysis........................................................................................................2

Supplementary Table S1. Baseline demographic characteristics for the parents/guardians..........................................................................................................4

Supplementary Table S2. Adherence to allocated treatment and treatment fidelity...........................................................................................................................5

Supplementary Table S3. Descriptives of secondary outcomes of teacher ratings and of suicidal ideation at baseline, week 4, and 6-months follow-up..............................................6

Supplementary Table S4. Parent reported and child reported side effects scale from randomisation to week 4..................................................................................................7

Supplementary Table S5. Overall adverse events category by group from randomization to month 6.........................................................................................................................12

Supplementary Table S6. Additional adverse events categories defined from free text (not mutually exclusive from original categories)..........................................................................13

Supplementary Table S7. Post-hoc analysis of primary outcome ADHD-RS total score at week 4 in participants aged between 8-12 years only......................................................................13

Supplementary Table S8. Post-hoc analysis of secondary outcome of Mind Excessive Wandering Scale (MEWS) at week 4 in participants aged 14-18 years.................................13

Supplementary Table S9. Post-hoc analysis of primary outcome ADHD-RS total score at week 4 in male participants......................................................................................................14

Supplementary Table S10. Post-hoc analysis of primary outcome ADHD-RS total score at week 4 in female participants...............................................................................................14

References........................................................................................................15

## Statistical analysis

Methods for the analysis follow the ATTENS Statistical Analysis Plan (SAP) v1.0 17/10/2023 which is a supplement to the protocol paper[1]. Below is a brief summary of the methods and a description of where any methods differ from what is stated in the SAP.

All main analyses used the intention to treat population, i.e., all randomised participants were included, and included per the group they were randomised to, targeting an estimand comparing real TNS to sham TNS regardless of discontinuation for any reason (treatment policy strategy). No other intercurrent events were observed.

The primary analysis model used a longitudinal linear mixed model with continuous time as a covariate using actual time of assessments and an interaction between time and trial arm to estimate effects at week 1, 2,3 and 4 using post-estimation. Per the SAP, continuous time was used as the assumption of a linear trend in the outcome over time did not appear to be violated. A random intercept was included as well as a random slope over time. Baseline ADHD-RS score and the stratification factors (site, age (8-13.5; 13.6-19), gender (Male; Female), and medication (On Medication; Off Medication/Medication naïve) were included as prognostic covariates. For the secondary endpoint at 6 months, a separate longitudinal model was used, a mixed model for repeated measures (MMRM) with categorical time, an interaction between categorical time and trial arm and heterogeneous error terms over time, with the same stratification factors included as covariates.

Secondary outcomes used the same model as above for the secondary ADHD-RS endpoint (a MMRM model with categorical time) where secondary outcomes were measured at 4 weeks and at 6 months. The objective hyperactivity outcome was only measured at 4 weeks, and therefore linear regression was used for this outcome, but otherwise the model used the same prognostic covariates.

For all outcome measures we report adjusted mean differences with 95% confidence intervals as well as Cohen's d calculated using the pooled baseline standard deviation of each measure.

Given that the percentage of missing data was extremely high, teacher rated outcomes of ADHD-RS and Conners were not analysed formally, and instead we have only presented them descriptively at week 4 (Table S6); any analysis would be underpowered and interpretation highly subject to potential bias from data being missing not at random. Similarly, we did not analyse the CSRSS suicidal ideation score due to the lack of variation in scores and instead we have only reported it descriptively at week 4 and at month 6 (Table S6); 98% of participants had a score of 0, indicating no suicidal ideation, and there was no score reported over 2 (out of 5). There would therefore be very low power for such analysis.

The amount of missing data in the primary outcome, as well as for many of the other secondary outcomes we analysed was very low, less than 5% (a few secondary outcomes had ~10% missingness). We considered this amount of missing data to be ignorable and it would have negligible effect on the results, as is a commonly applied "rule-of-thumb"[2]. As such, we did not carry out sensitivity analyses for missingness or checking of missingness predictors (under MAR assumption) pre-specified in the SAP.

Where baseline values were missing for an outcome, we used a baseline indicator method to include participants with missing baseline values in the model. This was done by setting

the missing baseline value to a fixed value (here we used 0.1) and including a binary indicator of missingness as an additional covariate.

As per the SAP, a separate analysis was carried out to estimate efficacy of the intervention in compliers for the primary outcome at week 4 (a complier average causal effect), with the definition of compliance being adherence to intervention (per Table S4) and excluding other protocol violators. There were no additional protocol violators, so the principal strata of compliers reflect adherence to the intervention only. This Complier Average Causal Effect (CACE) analysis was carried out using an instrumental variable linear mixed modelling approach in Stata (using *xtivreg*) with the linear mixed modelling component reflecting the same model as the primary outcome analysis model.

As there was little variance in compliance to the intervention as measured by hours and nights using the device, we did not carry out any exploratory analyses examining the dose-response relationship.

We pre-specified in the SAP that we would carry out a sensitivity analysis to examine the impact of sibling pairs if >20% of randomised participants were siblings. There were 8 sibling pairs, constituting 16 participants and therefore 10.7% of the randomised sample, so we did not carry out this sensitivity analysis.

Subgroup analyses were also carried out on the primary outcome as per the SAP, to estimate the effects in subgroups of those on stable medication and those off medication/medication naive. These analyses used the same model as the primary analysis but included in addition interaction terms between trial arm and the subgroup, time and the subgroup and a three-way interaction between trial arm, time, and subgroup. The estimated effects at week 4 in each subgroup were then extracted using post-estimation.

Additional post-hoc analyses not specified in the SAP were carried out following initial discussion of the results or as per journal requirements:

1. ADHD-RS outcome results in children aged 12 years or below 12 years only – this uses the same primary analysis model but with the sample restricted to children aged 12 or under 12 in order to replicate the analysis of the previously published pilot study[3].
2. MEWS outcome results in adolescents aged 14-18 years only – this uses the same secondary analysis model as for the MEWS, but with the sample restricted to adolescents 14 years or over. This additional analysis was done as it was thought that younger children below age 14 had difficulties understanding the questions.
3. ADHD-RS outcome results in male and female (sex at birth) participants – this uses the same primary analysis model but with the sample restricted to male participants or female participants. This analysis was done to comply with SAGER guidelines.

**Table S1.** Baseline demographic characteristics for the parents/guardians.

| Baseline characteristics (n, %) | Real TNS (n=75) | Sham TNS (n=75) | Overall (n=150) |
|---|---|---|---|
| **Parent/Guardian sex at birth** | | | |
| Male | 7 (9.3) | 6 (8.0) | 13 (8.7) |
| Female | 68 (90.7) | 69 (92.0) | 137 (91.3) |
| **Parent Relationship to Child** | | | |
| Parent | 74 (98.7) | 75 (100.0) | 149 (99.3) |
| Stepparent | 0 (0.0) | 0 (0.0) | 0 (0.0) |
| Foster parent/guardian | 1 (1.3) | 0 (0.0) | 1 (0.7) |
| **Highest education level completed by parent-guardian** | | | |
| Primary School (SATS) | 0 (0.0) | 0 (0.0) | 0 (0.0) |
| Secondary School (GCSE) | 6 (8.0) | 3 (4.0) | 9 (6.0) |
| Further education (A-level/ IB/ NVQ/ BTEC) | 18 (24.0) | 15 (20.0) | 33 (22.0) |
| Higher Education (University degree) | 29 (38.7) | 30 (40.0) | 59 (39.3) |
| Postgraduate Education (Masters, PhD) | 22 (29.3) | 27 (36.0) | 49 (32.7) |
| **Total family income (gross income before taxes and other deductions)** | | | |
| None - on benefits | 1 (1.3) | 1 (1.3) | 2 (1.3) |
| Up to £20,000 | 4 (5.3) | 3 (4.0) | 7 (4.7) |
| Between £20,001- £35,000 | 5 (6.7) | 11 (14.7) | 16 (10.7) |
| Between £35,001-£55,000 | 15 (20.0) | 9 (12.0) | 24 (16.0) |
| Above £55,001 | 50 (66.7) | 51 (68.0) | 101 (67.3) |
| **Parental home ownership** | | | |
| No | 17 (22.7) | 16 (21.3) | 33 (22.0) |
| Yes | 58 (77.3) | 59 (78.7) | 117 (78.0) |
| Number of people in the home (Mean (SD)) | 4.1 (0.9) | 3.9 (1.1) | 4.0 (1.0) |
| Number of bedrooms in the home (Mean (SD)) | 3.7 (1.1) | 3.7 (1.0) | 3.7 (1.1) |
| **Index of Multiple Deprivation** | | | |
| **1** | 2 (2.7) | 2 (2.7) | 4 (2.7) |
| **2** | 3 (4.1) | 2 (2.7) | 5 (3.4) |
| **3** | 8 (11.0) | 5 (6.8) | 13 (8.9) |
| **4** | 9 (12.3) | 8 (11.0) | 17 (11.6) |
| **5** | 7 (9.6) | 5 (6.8) | 12 (8.2) |
| **6** | 6 (8.2) | 8 (11.0) | 14 (9.6) |
| **7** | 5 (6.8) | 11 (15.1) | 16 (11.0) |
| **8** | 9 (12.3) | 9 (12.3) | 18 (12.3) |
| **9** | 8 (11.0) | 7 (9.6) | 15 (10.3) |
| **10** | 16 (21.9) | 16 (21.9) | 32 (21.9) |

**Note.** Categorical variables are presented as the number of participants, with the percentage in parentheses. Continuous variables are reported as mean (standard deviation). The Index of Multiple Deprivation shows the number and percentage of participants from 1 (most deprived area) to 10 (least deprived area).

**Table S2.** Adherence to allocated treatment and treatment fidelity.

| Adherence variable | Real TNS | Sham TNS | Overall |
|---|---|---|---|
| **Permanent discontinuation? (n, %)** | | | |
| Yes | 6 (8.0) | 3 (4.0) | 9 (6.0) |
| **Used the device >=1hour for >=17 nights (n, %)** | | | |
| Yes | 68 (90.7) | 72 (96.0) | 140 (93.3) |
| No | 7 (9.3) | 3 (4.0) | 10 (6.7) |
| **Main permanent discontinuation reason (n, %)** | | | |
| Problems with sleep/unable to sleep | 1 (16.7) | 0 (0.0) | 1 (11.1) |
| Other adverse event/side effect | 3 (50.0) | 2 (66.7) | 5 (55.6) |
| Other | 2 (33.3) | 1 (33.3) | 3 (33.3) |
| **Other Reason for discontinuation (n, %)** | | | |
| No specific reason given - child did not want to continue | 1 (50.0) | 0 (0.0) | 1 (33.3) |
| Participant did not come to appointments | 1 (50.0) | 1 (100.0) | 2 (66.7) |
| Total number of nights of device use (Mean, SD) | 25.2 (6.6) | 26.6 (3.0) | 25.9 (5.2) |
| Total number of hours of device use (Mean, SD) | 206.4 (74.0) | 229.9 (47.6) | 218.1 (63.1) |
| Average number of hours of device use per night (out of nights used) (Mean, SD) | 8.9 (1.3) [Range: 0.7-6.2] | 9.1(1.1) [Range:1-7.8] | 9.0 (1.2) [Range: 0.7-6.2] |
| Average device nightly setting-mA (Mean, SD) * | 2.3 (1.2) | 4.0 (1.6) | 3.1 (1.6) |

**Note.** *Device settings can take values from 0-10mA.Mean nightly settings were higher in the sham arm; this was expected as the frequency is lower in the sham, therefore participants would need a higher setting to feel the stimulation. mA=milliampere.

**Table S3.** Descriptives of secondary outcomes of teacher ratings and of suicidal ideation at baseline, week 4, and 6-months follow-up.

| **Baseline values** | **Real TNS** | | **Sham TNS** | |
|---|---|---|---|---|
| | **N** | **Mean (SD)** | **N** | **Mean (SD)** |
| **Teacher-rated ADHD symptoms - Conners Teacher Rating Scale- Inattention** | 26 | 69.9 (11.9) | 17 | 70.6 (12.6) |
| **Teacher-rated ADHD symptoms - Conners Teacher Rating Scale- Hyperactivity/Impulsiveness** | 26 | 69.0 (13.8) | 17 | 73.8 (15.1) |
| **Teacher rated ADHD-RS total score** | 25 | 23.3 (11.5) | 16 | 22.7 (12.4) |
| **Columbia Suicide Ideation Score (child rated)** | 75 | | 75 | |
| Low risk (n, %) | | 74 (98.7) | | 74 (98.7) |
| Moderate risk (n, %) | | 1 (1.3) | | 1 (1.3) |
| **4 weeks values** | | | | |
| **Teacher-rated ADHD symptoms - Conners Teacher Rating Scale- Inattention** | 16 | 72.6 (10.1) | 14 | 64.9 (12.2) |
| **Teacher-rated ADHD symptoms - Conners Teacher Rating Scale Hyperactivity/Impulsiveness** | 16 | 75.9 (14.2) | 15 | 66.0 (16.6) |
| **Teacher rated ADHD-RS total score** | 15 | 29.2 (12.8) | 13 | 20.4 (15.2) |
| **Columbia Suicide Ideation Score (child rated)** | 73 | | 74 | |
| Low risk (n, %) | | 73 (100.0) | | 74 (100.0) |
| Moderate risk (n, %) | | 0 (0.0) | | 0 (0.0) |
| **6 months values** | | | | |
| **Teacher rated ADHD-RS total score** | 7 | 12.0 (9.3) | 6 | 16.7 (6.5) |
| **Columbia Suicide Ideation Score (child rated)** | 71 | | 73 | |
| Low risk (n, %) | | 71 (100.0) | | 73(100) |
| Moderate risk (n, %) | | 0 (0.0) | | 0 (0.0) |

**Note.** ADHD-RS = ADHD rating scale. Columbia Suicide Ideation scores were calculated as follows: Low Risk: No suicidal ideation or behaviours reported; Moderate Risk: Suicidal thoughts with some intent or planning but no action taken. No teachers completed the Conners Teacher Rating Scale at 6 months.

**Table S4.** Parent reported and child reported side effects scale from randomisation to week 4**.**

| Side effects reported from randomisation to week 4* | Child reported | | Parent reported | |
|---|---|---|---|---|
| | Real TNS | Sham TNS | Real TNS | Sham TNS |
| **Trouble sleeping (N, %)** | | | | |
| None | 34 (45.3) | 31 (41.3) | 27 (36.0) | 30 (40.0) |
| Mild | 20 (26.7) | 19 (25.3) | 24 (32.0) | 25 (33.3) |
| Moderate | 14 (18.7) | 22 (29.3) | 17 (22.7) | 19 (25.3) |
| Severe | 7 (9.3) | 3 (4.0) | 7 (9.3) | 1 (1.3) |
| **Nightmares/ sleep disturbance (N, %)** | | | | |
| None | 59 (78.7) | 64 (85.3) | 57 (76.0) | 60 (80.0) |
| Mild | 10 (13.3) | 7 (9.3) | 10 (13.3) | 13 (17.3) |
| Moderate | 3 (4.0) | 2 (2.7) | 6 (8.0) | 1 (1.3) |
| Severe | 3 (4.0) | 2 (2.7) | 2 (2.7) | 1 (1.3) |
| **Drowsy/ Sleepy (N, %)** | | | | |
| None | 36 (48.0) | 34 (45.3) | 33 (44.0) | 36 (48.0) |
| Mild | 17 (22.7) | 16 (21.3) | 22 (29.3) | 22 (29.3) |
| Moderate | 15 (20.0) | 18 (24.0) | 15 (20.0) | 15 (20.0) |
| Severe | 7 (9.3) | 7 (9.3) | 5 (6.7) | 2 (2.7) |
| **Feeling nervous/hyper (N, %)** | | | | |
| None | 38 (50.7) | 37 (49.3) | 25 (33.3) | 29 (38.7) |
| Mild | 23 (30.7) | 13 (17.3) | 24 (32.0) | 20 (26.7) |
| Moderate | 10 (13.3) | 17 (22.7) | 21 (28.0) | 18 (24.0) |
| Severe | 4 (5.3) | 8 (10.7) | 5 (6.7) | 8 (10.7) |
| **Weakness/ Fatigue (N, %)** | | | | |
| None | 52 (69.3) | 46 (61.3) | 52 (69.3) | 49 (65.3) |
| Mild | 16 (21.3) | 14 (18.7) | 15 (20.0) | 20 (26.7) |
| Moderate | 3 (4.0) | 10 (13.3) | 6 (8.0) | 5 (6.7) |
| Severe | 4 (5.3) | 5 (6.7) | 2 (2.7) | 1 (1.3) |
| **Feeling strange/unreal (N, %)** | | | | |
| None | 63 (84.0) | 66 (88.0) | 67 (89.3) | 71 (94.7) |
| Mild | 9 (12.0) | 7 (9.3) | 7 (9.3) | 3 (4.0) |
| Moderate | 2 (2.7) | 1 (1.3) | 0 (0.0) | 1 (1.3) |
| Severe | 1 (1.3) | 1 (1.3) | 1 (1.3) | 0 (0.0) |
| **Hearing /seeing things (N, %)** | | | | |
| None | 72 (96.0) | 71 (94.7) | 74 (98.7) | 73 (97.3) |
| Mild | 2 (2.7) | 3 (4.0) | 1 (1.3) | 1 (1.3) |
| Moderate | 1 (1.3) | 0 (0.0) | 0 (0.0) | 1 (1.3) |
| Severe | 0 (0.0) | 1 (1.3) | 0 (0.0) | 0 (0.0) |
| **Numbness or tingling (N, %)** | | | | |
| None | 57 (76.0) | 60 (80.0) | 67 (89.3) | 73 (97.3) |
| Mild | 12 (16.0) | 14 (18.7) | 7 (9.3) | 1 (1.3) |

| | | | | |
|---|---|---|---|---|
| Moderate | 6 (8.0) | 1 (1.3) | 1 (1.3) | 1 (1.3) |
| Severe | 0 (0.0) | 0 (0.0) | 0 (0.0) | 0 (0.0) |
| **Dizziness or tingling (N, %)** | | | | |
| None | 63 (84.0) | 63 (84.0) | 67 (89.3) | 67 (89.3) |
| Mild | 11 (14.7) | 10 (13.3) | 8 (10.7) | 5 (6.7) |
| Moderate | 1 (1.3) | 2 (2.7) | 0 (0.0) | 3 (4.0) |
| Severe | 0 (0.0) | 0 (0.0) | 0 (0.0) | 0 (0.0) |
| **Headache (N, %)** | | | | |
| None | 35 (46.7) | 34 (45.3) | 37 (49.3) | 36 (48.0) |
| Mild | 23 (30.7) | 22 (29.3) | 24 (32.0) | 28 (37.3) |
| Moderate | 13 (17.3) | 16 (21.3) | 11 (14.7) | 8 (10.7) |
| Severe | 4 (5.3) | 3 (4.0) | 3 (4.0) | 3 (4.0) |
| **Blurred vision (N, %)** | | | | |
| None | 67 (89.3) | 69 (92.0) | 72 (96.0) | 73 (97.3) |
| Mild | 7 (9.3) | 5 (6.7) | 2 (2.7) | 2 (2.7) |
| Moderate | 0 (0.0) | 1 (1.3) | 0 (0.0) | 0 (0.0) |
| Severe | 1 (1.3) | 0 (0.0) | 1 (1.3) | 0 (0.0) |
| **Ringing in ears or trouble hearing (N, %)** | | | | |
| None | 58 (77.3) | 62 (82.7) | 66 (88.0) | 72 (96.0) |
| Mild | 14 (18.7) | 11 (14.7) | 7 (9.3) | 3 (4.0) |
| Moderate | 3 (4.0) | 2 (2.7) | 2 (2.7) | 0 (0.0) |
| Severe | 0 (0.0) | 0 (0.0) | 0 (0.0) | 0 (0.0) |
| **Stuffy nose (N, %)** | | | | |
| None | 42 (56.0) | 31 (41.3) | 46 (61.3) | 41 (54.7) |
| Mild | 21 (28.0) | 22 (29.3) | 20 (26.7) | 22 (29.3) |
| Moderate | 5 (6.7) | 15 (20.0) | 5 (6.7) | 6 (8.0) |
| Severe | 7 (9.3) | 7 (9.3) | 4 (5.3) | 6 (8.0) |
| **Dry mouth (N, %)** | | | | |
| None | 51 (68.0) | 55 (73.3) | 64 (85.3) | 66 (88.0) |
| Mild | 17 (22.7) | 13 (17.3) | 9 (12.0) | 6 (8.0) |
| Moderate | 5 (6.7) | 4 (5.3) | 1 (1.3) | 3 (4.0) |
| Severe | 2 (2.7) | 3 (4.0) | 1 (1.3) | 0 (0.0) |
| **Drooling or increased salivation (N, %)** | | | | |
| None | 68 (90.7) | 69 (92.0) | 72 (96.0) | 72 (96.0) |
| Mild | 6 (8.0) | 5 (6.7) | 1 (1.3) | 2 (2.7) |
| Moderate | 1 (1.3) | 1 (1.3) | 2 (2.7) | 1 (1.3) |
| Severe | 0 (0.0) | 0 (0.0) | 0 (0.0) | 0 (0.0) |
| **Muscle cramps or stiffness (N, %)** | | | | |
| None | 57 (76.0) | 54 (72.0) | 66 (88.0) | 64 (85.3) |
| Mild | 12 (16.0) | 15 (20.0) | 7 (9.3) | 6 (8.0) |
| Moderate | 5 (6.7) | 3 (4.0) | 1 (1.3) | 3 (4.0) |
| Severe | 1 (1.3) | 3 (4.0) | 1 (1.3) | 2 (2.7) |
| **Muscle twitching or movements (N, %)** | | | | |

| | | | | |
|---|---|---|---|---|
| None | 61 (81.3) | 63 (84.0) | 71 (94.7) | 67 (89.3) |
| Mild | 12 (16.0) | 10 (13.3) | 4 (5.3) | 6 (8.0) |
| Moderate | 2 (2.7) | 2 (2.7) | 0 (0.0) | 2 (2.7) |
| Severe | 0 (0.0) | 0 (0.0) | 0 (0.0) | 0 (0.0) |
| **Tremor or shakiness (N, %)** | | | | |
| None | 66 (88.0) | 67 (89.3) | 74 (98.7) | 74 (98.7) |
| Mild | 6 (8.0) | 7 (9.3) | 0 (0.0) | 0 (0.0) |
| Moderate | 3 (4.0) | 1 (1.3) | 1 (1.3) | 1 (1.3) |
| Severe | 0 (0.0) | 0 (0.0) | 0 (0.0) | 0 (0.0) |
| **Slurred speech (N, %)** | | | | |
| None | 69 (92.0) | 69 (92.0) | 74 (98.7) | 75 (100.0) |
| Mild | 6 (8.0) | 5 (6.7) | 1 (1.3) | 0 (0.0) |
| Moderate | 0 (0.0) | 1 (1.3) | 0 (0.0) | 0 (0.0) |
| Severe | 0 (0.0) | 0 (0.0) | 0 (0.0) | 0 (0.0) |
| **Heartbeat rapid or pounding (N, %)** | | | | |
| None | 64 (85.3) | 62 (82.7) | 69 (92.0) | 71 (94.7) |
| Mild | 7 (9.3) | 10 (13.3) | 5 (6.7) | 3 (4.0) |
| Moderate | 4 (5.3) | 3 (4.0) | 1 (1.3) | 1 (1.3) |
| Severe | 0 (0.0) | 0 (0.0) | 0 (0.0) | 0 (0.0) |
| **Trouble catching breath or hyperventilation (N, %)** | | | | |
| None | 68 (90.7) | 66 (88.0) | 72 (96.0) | 68 (90.7) |
| Mild | 5 (6.7) | 8 (10.7) | 3 (4.0) | 6 (8.0) |
| Moderate | 2 (2.7) | 1 (1.3) | 0 (0.0) | 1 (1.3) |
| Severe | 0 (0.0) | 0 (0.0) | 0 (0.0) | 0 (0.0) |
| **Chest pain (N, %)** | | | | |
| None | 67 (89.3) | 66 (88.0) | 71 (94.7) | 72 (96.0) |
| Mild | 7 (9.3) | 8 (10.7) | 3 (4.0) | 2 (2.7) |
| Moderate | 1 (1.3) | 1 (1.3) | 1 (1.3) | 1 (1.3) |
| Severe | 0 (0.0) | 0 (0.0) | 0 (0.0) | 0 (0.0) |
| **Nausea or vomiting (N, %)** | | | | |
| None | 69 (92.0) | 63 (84.0) | 69 (92.0) | 66 (88.0) |
| Mild | 3 (4.0) | 9 (12.0) | 4 (5.3) | 7 (9.3) |
| Moderate | 2 (2.7) | 3 (4.0) | 2 (2.7) | 2 (2.7) |
| Severe | 1 (1.3) | 0 (0.0) | 0 (0.0) | 0 (0.0) |
| **Stomach or abdominal discomfort (N, %)** | | | | |
| None | 53 (70.7) | 60 (80.0) | 58 (77.3) | 56 (74.7) |
| Mild | 16 (21.3) | 6 (8.0) | 12 (16.0) | 11 (14.7) |
| Moderate | 5 (6.7) | 7 (9.3) | 5 (6.7) | 8 (10.7) |
| Severe | 1 (1.3) | 2 (2.7) | 0 (0.0) | 0 (0.0) |
| **Constipation (N, %)** | | | | |
| None | 74 (98.7) | 68 (90.7) | 75 (100.0) | 67 (89.3) |
| Mild | 1 (1.3) | 5 (6.7) | 0 (0.0) | 7 (9.3) |
| Moderate | 0 (0.0) | 2 (2.7) | 0 (0.0) | 1 (1.3) |

| | | | | |
|---|---|---|---|---|
| Severe | 0 (0.0) | 0 (0.0) | 0 (0.0) | 0 (0.0) |
| **Diarrhoea (N, %)** | | | | |
| None | 69 (92.0) | 65 (86.7) | 68 (90.7) | 66 (88.0) |
| Mild | 5 (6.7) | 5 (6.7) | 6 (8.0) | 6 (8.0) |
| Moderate | 0 (0.0) | 4 (5.3) | 0 (0.0) | 3 (4.0) |
| Severe | 1 (1.3) | 1 (1.3) | 1 (1.3) | 0 (0.0) |
| **Frequent need to urinate (N, %)** | | | | |
| None | 69 (92.0) | 60 (80.0) | 71 (94.7) | 69 (92.0) |
| Mild | 4 (5.3) | 8 (10.7) | 2 (2.7) | 2 (2.7) |
| Moderate | 1 (1.3) | 7 (9.3) | 1 (1.3) | 4 (5.3) |
| Severe | 1 (1.3) | 0 (0.0) | 1 (1.3) | 0 (0.0) |
| **Sweating excessively (N, %)** | | | | |
| None | 65 (86.7) | 63 (84.0) | 65 (86.7) | 69 (92.0) |
| Mild | 8 (10.7) | 11 (14.7) | 8 (10.7) | 6 (8.0) |
| Moderate | 1 (1.3) | 1 (1.3) | 2 (2.7) | 0 (0.0) |
| Severe | 1 (1.3) | 0 (0.0) | 0 (0.0) | 0 (0.0) |
| **Appetite decreased (N, %)** | | | | |
| None | 59 (78.7) | 51 (68.0) | 57 (76.0) | 52 (69.3) |
| Mild | 10 (13.3) | 11 (14.7) | 9 (12.0) | 14 (18.7) |
| Moderate | 5 (6.7) | 11 (14.7) | 7 (9.3) | 7 (9.3) |
| Severe | 1 (1.3) | 2 (2.7) | 2 (2.7) | 2 (2.7) |
| **Appetite increased (N, %)** | | | | |
| None | 51 (68.0) | 48 (64.0) | 53 (70.7) | 50 (66.7) |
| Mild | 14 (18.7) | 14 (18.7) | 16 (21.3) | 9 (12.0) |
| Moderate | 7 (9.3) | 11 (14.7) | 5 (6.7) | 15 (20.0) |
| Severe | 3 (4.0) | 2 (2.7) | 1 (1.3) | 1 (1.3) |
| **Skin rash or allergy (N, %)** | | | | |
| None | 66 (88.0) | 62 (82.7) | 66 (88.0) | 64 (85.3) |
| Mild | 7 (9.3) | 9 (12.0) | 8 (10.7) | 9 (12.0) |
| Moderate | 2 (2.7) | 4 (5.3) | 1 (1.3) | 2 (2.7) |
| Severe | 0 (0.0) | 0 (0.0) | 0 (0.0) | 0 (0.0) |
| **Difficulties finding words (N, %)** | | | | |
| None | 59 (78.7) | 65 (86.7) | 68 (90.7) | 64 (85.3) |
| Mild | 9 (12.0) | 9 (12.0) | 5 (6.7) | 7 (9.3) |
| Moderate | 5 (6.7) | 1 (1.3) | 2 (2.7) | 3 (4.0) |
| Severe | 2 (2.7) | 0 (0.0) | 0 (0.0) | 1 (1.3) |
| **Apathy/emotional indifference (N, %)** | | | | |
| None | 58 (77.3) | 59 (78.7) | 62 (82.7) | 56 (74.7) |
| Mild | 13 (17.3) | 10 (13.3) | 8 (10.7) | 14 (18.7) |
| Moderate | 3 (4.0) | 3 (4.0) | 2 (2.7) | 5 (6.7) |
| Severe | 1 (1.3) | 3 (4.0) | 3 (4.0) | 0 (0.0) |
| **Bruising (N, %)** | | | | |
| None | 69 (92.0) | 68 (90.7) | 74 (98.7) | 69 (92.0) |

| | | | | |
|---|---|---|---|---|
| Mild | 5 (6.7) | 6 (8.0) | 1 (1.3) | 6 (8.0) |
| Moderate | 1 (1.3) | 1 (1.3) | 0 (0.0) | 0 (0.0) |
| Severe | 0 (0.0) | 0 (0.0) | 0 (0.0) | 0 (0.0) |
| **Hair thinning/loss (N, %)** | | | | |
| None | 74 (98.7) | 74 (98.7) | 75 (100.0) | 75 (100.0) |
| Mild | 0 (0.0) | 1 (1.3) | 0 (0.0) | 0 (0.0) |
| Moderate | 1 (1.3) | 0 (0.0) | 0 (0.0) | 0 (0.0) |
| Severe | 0 (0.0) | 0 (0.0) | 0 (0.0) | 0 (0.0) |
| **Hot flashes (N, %)** | | | | |
| None | 65 (86.7) | 64 (85.3) | 73 (97.3) | 67 (89.3) |
| Mild | 8 (10.7) | 9 (12.0) | 2 (2.7) | 7 (9.3) |
| Moderate | 1 (1.3) | 2 (2.7) | 0 (0.0) | 1 (1.3) |
| Severe | 1 (1.3) | 0 (0.0) | 0 (0.0) | 0 (0.0) |
| **Clenching of teeth at night (N, %)** | | | | |
| None | 68 (90.7) | 68 (90.7) | 63 (84.0) | 70 (93.3) |
| Mild | 5 (6.7) | 4 (5.3) | 12 (16.0) | 3 (4.0) |
| Moderate | 2 (2.7) | 2 (2.7) | 0 (0.0) | 2 (2.7) |
| Severe | 0 (0.0) | 1 (1.3) | 0 (0.0) | 0 (0.0) |

**Note.** *Cumulative over the 4 weeks and the highest severity reported over that time point

**Table S5.** Overall adverse events category by group from randomization to month 6.

| AE Category | Real TNS | | | | Sham TNS | | | |
|---|---|---|---|---|---|---|---|---|
| | AES | | ADES | | AES | | ADES | |
| | N events | N with event (%) | N events | N with event (%) | N events | N with event (%) | N events | N with event (%) |
| Anxiety or worrying | 0 | 0 (0.0) | 2 | 1 (1.3) | 1 | 1 (1.3) | 2 | 2 (2.7) |
| Tearfulness, sadness, or depression | 1 | 1 (1.3) | 1 | 1 (1.3) | 3 | 3 (4.0) | 5 | 5 (6.7) |
| Tiredness, demotivation, joylessness | 0 | 0 (0.0) | 3 | 3 (4.0) | 0 | 0 (0.0) | 1 | 1 (1.3) |
| Withdrawal or less socializing (less interested in peers) | 0 | 0 (0.0) | 0 | 0 (0.0) | 0 | 0 (0.0) | 0 | 0 (0.0) |
| Grumpiness and irritability | 1 | 1 (1.3) | 3 | 3 (4.0) | 2 | 1 (1.3) | 5 | 4 (5.3) |
| Twitching or ticks (eye blinking, head tics) | 0 | 0 (0.0) | 0 | 0 (0.0) | 0 | 0 (0.0) | 1 | 1 (1.3) |
| Scratching himself/ herself, biting nails or lips more | 0 | 0 (0.0) | 0 | 0 (0.0) | 0 | 0 (0.0) | 1 | 1 (1.3) |
| Headaches | 2 | 2 (2.7) | 18 | 16 (21.3) | 1 | 1 (1.3) | 15 | 13 (17.3) |
| Stomach aches | 1 | 1 (1.3) | 0 | 0 (0.0) | 2 | 2 (2.7) | 0 | 0 (0.0) |
| Lack of appetite | 0 | 0 (0.0) | 1 | 1 (1.3) | 0 | 0 (0.0) | 1 | 1 (1.3) |
| Problems falling asleep or sleep problems | 0 | 0 (0.0) | 17 | 15 (20.0) | 0 | 0 (0.0) | 8 | 7 (9.3) |
| Frustration | 0 | 0 (0.0) | 3 | 3 (4.0) | 0 | 0 (0.0) | 1 | 1 (1.3) |
| Lack of confidence | 0 | 0 (0.0) | 0 | 0 (0.0) | 0 | 0 (0.0) | 0 | 0 (0.0) |
| Distractibility | 0 | 0 (0.0) | 0 | 0 (0.0) | 0 | 0 (0.0) | 0 | 0 (0.0) |
| Other physiological event | 21 | 19 (25.3) | 12 | 10 (13.3) | 23 | 19 (25.3) | 6 | 4 (5.3) |
| Other psychological event | 4 | 3 (4.0) | 7 | 5 (6.7) | 3 | 3 (4.0) | 3 | 3 (4.0) |

**Note.** AE=Adverse Event; AES=Adverse Events; ADES=Adverse Device Effects.

**Table S6.** Additional adverse events categories defined from free text (not mutually exclusive from original categories).

| AE Category | Real TNS | | Sham TNS | |
|---|---|---|---|---|
| | N events | N with event (%) | N events | N with event (%) |
| School Related | 5 | 4 (5.3) | 3 | 3 (4.0) |
| Family or Friend Interaction | 1 | 1 (1.3) | 7 | 4 (5.3) |
| Worsening ADHD symptoms | 5 | 5 (6.7) | 3 | 3 (4.0) |

**Note.** AE=Adverse Event.

**Table S7.** Post-hoc analysis of primary outcome ADHD-RS total score at week 4 in participants aged between 8-12 years only.

| Visit | Real TNS | | | Sham TNS | | | aMD (95% CI) | p-value |
|---|---|---|---|---|---|---|---|---|
| Visit | N | Mean | SD | N | Mean | SD | | |
| Baseline | 41 | 35.2 | 9.9 | 38 | 37.3 | 9.6 | | |
| Week 1 | 41 | 27.9 | 11.6 | 38 | 26.5 | 10.9 | 2.59 (-1.11,6.29) | |
| Week 2 | 41 | 26.7 | 12.1 | 38 | 26.3 | 12.9 | 1.91 (-1.65,5.47) | |
| Week 3 | 41 | 25.3 | 12.2 | 38 | 25.6 | 11.4 | 1.23 (-2.54,5.01) | |
| Week 4 | 41 | 27.9 | 12.1 | 37 | 29.0 | 12.2 | 0.55 (-3.73,4.83) | 0.80 |

**Note.** aMD=Adjusted Mean Difference. P-values were calculated using two-sided z-tests from the linear mixed models as outlined in methods.

**Table S8.** Post-hoc analysis of secondary outcome of Mind Excessive Wandering Scale (MEWS) at week 4 in participants aged 14-18 years.

| Visit | Real TNS | | | Sham TNS | | | aMD (95% CI) | p-value |
|---|---|---|---|---|---|---|---|---|
| Visit | N | Mean | SD | N | Mean | SD | | |
| Baseline | 21 | 16.1 | 9.4 | 23 | 17.0 | 9.1 | | |
| Week 4 | 21 | 14.0 | 10.0 | 23 | 14.6 | 10.7 | 0.28 (-3.65,4.21) | 0.89 |

**Note.** aMD=Adjusted Mean Difference. P-values were calculated using two-sided z-tests from the linear mixed models as outlined in methods.

Table S9 and S10 below show the primary outcome disaggregated by sex at birth. At the week 4 primary endpoint, no significant group differences between real and sham TNS were observed in ADHD-RS total score in male participants (aMD = –0.96; 95% CI: –5.08 to 3.16; p = 0.65) or in female participants (aMD = 4.05; 95% CI: –1.51 to 9.62; p = 0.15).

**Table S9.** Post-hoc analysis of primary outcome ADHD-RS total score at week 4 in male participants.

| Visit | Real TNS | | | Sham TNS | | | aMD (95% CI) | p-value |
|---|---|---|---|---|---|---|---|---|
| | N | Mean | SD | N | Mean | SD | | |
| Baseline | 49 | 36.2 | 10.1 | 48 | 35.4 | 9.8 | | |
| Week 1 | 49 | 27.8 | 11.4 | 48 | 24.6 | 12.0 | 3.17 (-0.08,6.41) | |
| Week 2 | 48 | 27.2 | 12.6 | 48 | 24.1 | 12.5 | 1.79 (-1.41,4.99) | |
| Week 3 | 47 | 24.5 | 11.2 | 48 | 24.6 | 11.9 | 0.42 (-3.10,3.93) | |
| Week 4 | 47 | 26.3 | 11.6 | 47 | 27.3 | 12.1 | -0.96 (-5.08,3.16) | 0.65 |

**Note.** aMD=Adjusted Mean Difference. P-values were calculated using two-sided z-tests from the linear mixed models as outlined in methods.

**Table S10.** Post-hoc analysis of primary outcome ADHD-RS total score at week 4 in female participants.

| Visit | Real TNS | | | Sham TNS | | | aMD (95% CI) | p-value |
|---|---|---|---|---|---|---|---|---|
| | N | Mean | SD | N | Mean | SD | | |
| Baseline | 26 | 34.0 | 8.9 | 27 | 34.9 | 9.9 | | |
| Week 1 | 26 | 24.2 | 12.5 | 27 | 19.9 | 9.6 | 2.82 (-1.59,7.23) | |
| Week 2 | 26 | 22.0 | 12.2 | 27 | 20.8 | 11.9 | 3.23 (-1.12,7.58) | |
| Week 3 | 26 | 23.4 | 13.4 | 27 | 18.6 | 11.4 | 3.64 (-1.13,8.41) | |
| Week 4 | 26 | 25.6 | 13.7 | 27 | 21.0 | 11.9 | 4.05 (-1.51,9.62) | 0.15 |

**Note.** aMD=Adjusted Mean Difference. P-values were calculated using two-sided z-tests from the linear mixed models as outlined in methods.

## References

1. Rubia, K., *et al.* The efficacy of real versus sham external Trigeminal Nerve Stimulation (eTNS) in youth with Attention-Deficit/Hyperactivity Disorder (ADHD) over 4 weeks: a protocol for a multi-centre, double-blind, randomized, parallel-group, phase IIb study (ATTENS). *BMC Psychiatry* **24**, 326 (2024). https://doi.org/10.1186/s12888-024-05650-1
2. Jakobsen, J.C., Gluud, C., Wetterslev, J. & Winkel, P. When and how should multiple imputation be used for handling missing data in randomised clinical trials - a practical guide with flowcharts. *BMC Med Res Methodol* **17**, 162 (2017). https://doi.org/10.1186/s12874-017-0442-1
3. McGough, J.J., *et al.* Double-Blind, Sham-Controlled, Pilot Study of Trigeminal Nerve Stimulation for Attention-Deficit/Hyperactivity Disorder. *J Am Acad Child Adolesc Psychiatry* **58**, 403-411 e403 (2019)
