# Supplementary material for: External trigeminal nerve stimulation in youth with ADHD: a randomized, sham-controlled, phase 2b trial
Source: Nat Med. 2026 Jan 16;32(2):582–90. doi: 10.1038/s41591-025-04075-x (PMC12920111; doi:10.1038/s41591-025-04075-x)
Supplement: Supplementary file 2 — Reporting Summary [file 41591_2025_4075_MOESM2_ESM.pdf]

nature portfolio

Corresponding author(s): Katya Rubia
Last updated by author(s): Sep 10, 2025

Reporting Summary

Nature Portfolio wishes to improve the reproducibility of the work that we publish. This form provides structure for consistency and transparency in reporting. For further information on Nature Portfolio policies, see our Editorial Policies and the Editorial Policy Checklist.

Statistics

For all statistical analyses, confirm that the following items are present in the figure legend, table legend, main text, or Methods section.

n/a	Confirmed	
☐	☒	The exact sample size (*n*) for each experimental group/condition, given as a discrete number and unit of measurement
☐	☒	A statement on whether measurements were taken from distinct samples or whether the same sample was measured repeatedly
☐	☒	The statistical test(s) used AND whether they are one- or two-sided<br>*Only common tests should be described solely by name; describe more complex techniques in the Methods section.*
☐	☒	A description of all covariates tested
☐	☒	A description of any assumptions or corrections, such as tests of normality and adjustment for multiple comparisons
☐	☒	A full description of the statistical parameters including central tendency (e.g. means) or other basic estimates (e.g. regression coefficient) AND variation (e.g. standard deviation) or associated estimates of uncertainty (e.g. confidence intervals)
☐	☒	For null hypothesis testing, the test statistic (e.g. *F*, *t*, *r*) with confidence intervals, effect sizes, degrees of freedom and *P* value noted<br>*Give P values as exact values whenever suitable.*
☒	☐	For Bayesian analysis, information on the choice of priors and Markov chain Monte Carlo settings
☐	☒	For hierarchical and complex designs, identification of the appropriate level for tests and full reporting of outcomes
☐	☒	Estimates of effect sizes (e.g. Cohen's *d*, Pearson's *r*), indicating how they were calculated

Our web collection on statistics for biologists contains articles on many of the points above.

Software and code

Policy information about availability of computer code

Data collection	the MACRO electronic data capture (EDC) system (v4.15.0.116) was used to store clinical trial data. The Tobii Pro Eye Tracker Manager software (Tobii AB, Stockholm, Sweden,Tobii Pro Lab v1.207) was utilized to process pupillometry data. Computerized cognitive task data were processed through Microsoft EXCEL. Objective Hyperactivity data were processed through the Empatica E4 Manager software (Empatica Srl, Milan, Italy v2.0.3 (5119)).
Data analysis	Data analysis was conducted using Stata 18 (StataCorp LLC, v18.0) in accordance with a pre-specified statistical analysis plan. No custom or unpublished software was used.

For manuscripts utilizing custom algorithms or software that are central to the research but not yet described in published literature, software must be made available to editors and reviewers. We strongly encourage code deposition in a community repository (e.g. GitHub). See the Nature Portfolio guidelines for submitting code & software for further information.

Data

Policy information about availability of data

All manuscripts must include a data availability statement. This statement should provide the following information, where applicable:

- Accession codes, unique identifiers, or web links for publicly available datasets
- A description of any restrictions on data availability
- For clinical datasets or third party data, please ensure that the statement adheres to our policy

All data supporting the findings of this study have been deposited in the Figshare repository and are publicly available at https://doi.org/10.6084/m9.figshare.29414744.v1. Source data underlying the figures and tables presented in this paper are included in the Figshare repository. No custom code was generated or used in this study.

Research involving human participants, their data, or biological material

Policy information about studies with human participants or human data. See also policy information about sex, gender (identity/presentation), and sexual orientation and race, ethnicity and racism.

Reporting on sex and gender	Biological sex assigned at birth (male or female) was collected as reported by parents/guardians during eligibility assessments. Sex at birth was considered in the study design and was used both as a stratification factor during randomization and as a covariate in the primary and secondary statistical analyses. Overall, 97 male and 53 female participants were recruited. In the real TNS arm, 65.3% of participants were male and 34.7% were female, while in the sham TNS arm, 64.0% were male and 36.0% were female. In accordance with the Sex and Gender Equity in Research (SAGER) guidelines, we conducted post hoc analyses of the primary outcome disaggregated by sex at birth.
Reporting on race, ethnicity, or other socially relevant groupings	Ethnicity was self-reported by participants' parents or guardians at eligibility using standardized UK Census categories (e.g., White, Black/African/Caribbean/Black British, Asian/Asian British, Mixed/Multiple ethnic groups, and Other). The purpose of collecting this data was to assess the representativeness of the study sample. This variable was not used as a proxy for socioeconomic status, which was independently assessed using the Index of Multiple Deprivation. The sample was broadly representative of the UK general population, with 79.3% identifying as White and 20.7% from other ethnic groups.<br>To control for potential confounders, statistical analyses employed longitudinal linear mixed models with fixed effects for key covariates, including age group (8–13.5 years vs. 13.6–19 years), sex, site (King's College London vs. University of Southampton), baseline ADHD symptom severity (ADHD-RS score), and medication status (on stable stimulant medication vs. off/medication-naïve). These adjustments were specified a priori and implemented consistently across outcome models to reduce bias and improve the precision of treatment effect estimates.
Population characteristics	The study population consisted of 150 children and adolescents aged 8 to 18 years (mean age = 12.6 years, SD = 2.8) who met the diagnostic criteria for Attention-Deficit/Hyperactivity Disorder (ADHD) according to the DSM-5, as assessed using the semi-structured Kiddie Schedule for Affective Disorders and Schizophrenia (K-SADS). The sample included 64.7% males and 35.3% females. In terms of ADHD subtypes, 88.7% had the combined presentation, 10.7% had the inattentive presentation, and 0.7% had the hyperactive/impulsive presentation.Ethnically, the sample was 79.3% White, 10% Mixed or Multiple ethnic groups, 4.7% Asian or Asian British, 3.3% Black, African, Caribbean, or Black British, and 2.7% from other ethnic backgrounds. This distribution closely mirrors the general UK population, enhancing generalizability.Participants' IQ was within the typical range, with a mean Full Scale IQ (WASI FSIQ-4) of 107.6 (SD = 13.8). Comorbid Oppositional Defiant Disorder was present in 36% of participants, and Conduct Disorder in 2.7%. At baseline, 60.7% of participants were medication-naïve or off medication, and 39.3% were on stable stimulant medication.Participants were stratified at randomization by age (8–13.5 vs. 13.6–19 years), sex, site (King's College London or University of Southampton), and medication status. The cohort represented a diverse range of socioeconomic backgrounds as assessed by the Index of Multiple Deprivation (mean = 6.7, SD = 2.7).
Recruitment	Participants were recruited between September 2022 and November 2024 from public and private clinics in Greater London, Southampton, and Portsmouth; nationwide parent and ADHD support groups; general practitioners (GPs); the NHS Consent for Contact (C4C) research directory; and social media. To minimize selection bias and enhance external validity, recruitment efforts targeted both urban and suburban areas in London and Southampton and sought to include families from a range of socioeconomic backgrounds. Stratified randomization was employed to ensure balance across key variables (age, sex, site, and medication status). The use of broad eligibility criteria and the inclusion of both medicated and non-medicated participants further reduced the risk of sampling bias. To support engagement, participation incentives and reimbursement of travel expenses were provided. Children and their parents/carers gave both digital and written informed consent/assent. Participants were reimbursed for travel costs and received up to £350 for study completion (or up to £450 for those enrolled in the fMRI sub-study, which will be published separately).
Ethics oversight	The trial was approved by the West Midlands–Solihull NHS Research Ethics Committee (REC; Ref: 21/WN/0169) and the Medicines and Healthcare products Regulatory Agency (MHRA; Ref: CI/2022/0003/GB). It was conducted in accordance with the Declaration of Helsinki 1975 and is reported following CONSORT guidelines. Independent oversight of the trial was provided by a data monitoring committee and a trial steering committee.

Note that full information on the approval of the study protocol must also be provided in the manuscript.

Field-specific reporting

Please select the one below that is the best fit for your research. If you are not sure, read the appropriate sections before making your selection.

☒ Life sciences ☐ Behavioural & social sciences ☐ Ecological, evolutionary & environmental sciences

For a reference copy of the document with all sections, see nature.com/documents/nr-reporting-summary-flat.pdf

Life sciences study design

All studies must disclose on these points even when the disclosure is negative.

Sample size	The estimated sample size of 128 participants (64:64) was calculated using a baseline to post-treatment correlation of 0.5, 90% power, 5% type I error, and an anticipated effect size of 0.5 for a reduction in ADHD symptoms at 4 weeks. The number of participants was inflated to 150 (75:75) to account for a loss to follow-up rate of 15%.
Data exclusions	Data were analyzed using an intention-to-treat (ITT) approach, with all randomized participants included in the final analyses.
Replication	This study was a multi-centre, double-blind, randomized, sham-controlled, parallel-group, phase IIb RCT investigating the efficacy and safety of external trigeminal nerve stimulation (TNS) on core symptoms of ADHD and related clinical, cognitive, and physiological outcomes in youths with ADHD. It was conducted across two independent sites in the UK (King's College London and the University of Southampton), and It was designed as a confirmatory study following a previous pilot RCT conducted by another research group in the US (McGough et al., 2019). No experimental conditions were repeated in independent cohorts beyond this trial.<br>Reference:<br>McGough, J. J., Sturm, A., Cowen, J., Tung, K., Salgari, G. C., Leuchter, A. F., ... & Loo, S. K. (2019). Double-blind, sham-controlled, pilot study of trigeminal nerve stimulation for attention-deficit/hyperactivity disorder. Journal of the American Academy of Child & Adolescent Psychiatry, 58(4), 403-411.https://doi.org/10.1016/j.jaac.2018.11.013
Randomization	Randomization was done by minimization by sex (male/female), medication status (on medication; off medication/naïve), site (London, Southampton) and age (8–13.5 years; 13.6-19 years) using a validated, online, web-based system from King's Clinical Trials unit (KCTU).
Blinding	Participants, parents/carers, postdoctoral research associates, Principal Investigator, Co-Investigators, and analysts were blinded to treatment arm except for the trial manager and trial manager assistants, who trained participants/parents on the device use, but did not conduct research assessments and were prohibited from sharing the information with other team members. Analysts were blinded until after database lock. Blinding was assessed by a questionnaire administered to participants, parents/carers, and researchers after 1 and 4 weeks of TNS treatment.

Reporting for specific materials, systems and methods

We require information from authors about some types of materials, experimental systems and methods used in many studies. Here, indicate whether each material, system or method listed is relevant to your study. If you are not sure if a list item applies to your research, read the appropriate section before selecting a response.

Materials & experimental systems

n/a	Involved in the study	
☒	☐	Antibodies
☒	☐	Eukaryotic cell lines
☒	☐	Palaeontology and archaeology
☒	☐	Animals and other organisms
☐	☒	Clinical data
☒	☐	Dual use research of concern
☒	☐	Plants

Methods

n/a	Involved in the study	
☒	☐	ChIP-seq
☒	☐	Flow cytometry
☒	☐	MRI-based neuroimaging

Clinical data

Policy information about clinical studies

All manuscripts should comply with the ICMJE guidelines for publication of clinical research and a completed CONSORT checklist must be included with all submissions.

Clinical trial registration	Trial registration: ISRCTN82129325
Study protocol	The full trial protocol and the SAP has been published. See reference below. -Rubia, K., Johansson, L., Carter, B. et al. The efficacy of real versus sham external Trigeminal Nerve Stimulation (eTNS) in youth with Attention-Deficit/Hyperactivity Disorder (ADHD) over 4 weeks: a protocol for a multi-centre, double-blind, randomized, parallelgroup, phase IIb study (ATTENS). BMC Psychiatry 24, 326 (2024). https://doi.org/10.1186/s12888-024-05650-1

Data collection	Participant recruitment began in September 2022 and concluded in November 2024. Data were collected between September 2022 and March 2025 (6 months follow-up data collection) at two sites in the United Kingdom: King's College London (Institute of Psychiatry, Psychology and Neuroscience) and the University of Southampton (Centre for Innovation in Mental Health). Study assessments were conducted in research facilities within these universities, including laboratories for cognitive testing, physiological measurements, and behavioral assessments, to ensure standardized and controlled data collection.
Outcomes	The primary outcome measure was the investigator-scored, parent-rated ADHD-RS total score collected at eligibility, baseline and weekly throughout the four-week trial. Secondary outcome measures were collected at baseline, week 4, and at 6 months follow-up and included the following rating scales: teacher-rated ADHD-RS (school version) , Conners Teacher Rating Scale short form T-S , child-reported Strength and Difficulties Questionnaire (SDQ) , parent and child-reported Affective Reactivity Index (ARI) , parent and child-reported Child and Adolescent Anxiety and Depression scale (RCADS-25), child-reported Columbia Suicide Severity Rating Scale (C-SSRS) , child-reported Mind Excessively Wandering Scale (MEWS), parent-reported Sleep Disturbance Scale for Children (SDSC), and the investigator scored parent-rated ADHD-RS at 6 months follow-up. Vigilance (omission and commission errors) was assessed using the Mackworth Clock Task. Pupillometry data were recorded with the Tobii Pro Nano screen-based eye-tracking device (Tobii AB, Stockholm, Sweden) during a 1-minute resting condition and a cognitive task. Objective hyperactivity, defined as the composite score of both the intensity (g) and frequency (g) of movement, was assessed at baseline and week 4 using a 3-axis accelerometer embedded in the Empatica E4 wristband device (Empatica Srl, Milan, Italy). Other measures included an acceptability questionnaire filled out by participants and their parents/carers at the end of the treatment, side effects questionnaires and open-ended adverse event forms completed by participants and their parents/carers at baseline, week 4, and at 6 months follow-up. Blinding was assessed by a questionnaire administered to participants, parents/carers, and researchers after 1 and 4 weeks of TNS treatment.

Plants

Seed stocks	*Report on the source of all seed stocks or other plant material used. If applicable, state the seed stock centre and catalogue number. If plant specimens were collected from the field, describe the collection location, date and sampling procedures.*
Novel plant genotypes	*Describe the methods by which all novel plant genotypes were produced. This includes those generated by transgenic approaches, gene editing, chemical/radiation-based mutagenesis and hybridization. For transgenic lines, describe the transformation method, the number of independent lines analyzed and the generation upon which experiments were performed. For gene-edited lines, describe the editor used, the endogenous sequence targeted for editing, the targeting guide RNA sequence (if applicable) and how the editor was applied.*
Authentication	*Describe any authentication procedures for each seed stock used or novel genotype generated. Describe any experiments used to assess the effect of a mutation and, where applicable, how potential secondary effects (e.g. second site T-DNA insertions, mosiacism, off-target gene editing) were examined.*
